# Contribution of green turtles *Chelonia mydas* to total herbivore biomass in shallow tropical reefs of oceanic islands

**DOI:** 10.1371/journal.pone.0228548

**Published:** 2020-01-30

**Authors:** Luis Cardona, Patricia Campos, Adriana Velásquez-Vacca

**Affiliations:** IRBio and Department of Evolutionary Biology, Ecology and Environmental Science, Faculty of Biology, University of Barcelona, Barcelona, Spain; Australian Bureau of Agricultural and Resource Economics and Sciences, AUSTRALIA

## Abstract

Green turtles are megaherbivores with a key role in the dynamics of tropical seagrass meadows, but little is known about their relevance as herbivores in tropical reef habitats. We conducted underwater censuses of green turtles, herbivorous fishes and sea urchins in two distinct tropical regions: Fernando de Noronha (Western Atlantic Ocean) and the Hawaiian Archipelago (Central Pacific Ocean), to assess the contribution of green turtles to the total herbivore biomass in shallow reef habitats of tropical oceanic islands. Juvenile green turtles ranging 40–60 cm were observed at most of the surveyed sites, and hence, could be considered typical components of the shallow reef fauna of tropical oceanic islands. Furthermore, they were usually one of the most abundant species of roving herbivores in many of the sites surveyed. However, the biomass of green turtles was usually much lower than the aggregated biomass of fishes or sea urchins, which usually constituted most of the total herbivore biomass. Green turtles made a major contribution to the total herbivore biomass only in sheltered sites with low rugosity, low coral cover and high algal cover. Further investigation on the trophic redundancy between herbivores is required to assess the actual relevance of green turtles in reef ecosystems of oceanic islands, compared to herbivorous fishes and sea urchins, because different herbivores may target different algal resources and complementarity may be needed to maintain ecosystem functioning across large, naturally varied reefscapes.

## Introduction

Herbivory is a critical process in shallow marine ecosystems worldwide and changes in herbivore biomass may have profound effects on ecosystem structure [1–3]. Tropical reefs are not an exception and the foraging activity of sea urchins, fishes and, to a lesser extent, crabs, creates open spaces allowing the settlement of coral colonies [4–8]. Although the actual abundance of macroalgae [9] and the exact relevance of individual herbivore species in healthy tropical reefs is debated [4, 10–12], the existence of an abundant and diverse assemblage of herbivores is thought to increase coral reef resilience and create a buffer for natural and human induced disturbances [6, 7, 13].

Green turtles *Chelonia mydas* are megaherbivores occurring in tropical regions worldwide [14]. Some populations were decimated historically due to a combination of overharvesting, bycatch, loss or alteration of nesting habitat, degradation and loss of foraging habitat, and entanglement in or ingestion of marine debris [15]. Nevertheless, most populations are currently increasing thanks to conservation actions implemented during the past decades [16]. Recovery has been particularly successful in the Western South Atlantic and the Hawaiian Archipelago [17, 18], where subpopulations have recently been classified as not threatened by the International Union for Conservation of Nature [19, 20].

Recent evidence shows that green turtle grazing is a major structuring force in seagrass meadows once populations are rebuilt [3, 21, 22] and the same could be true in other habitats. Tropical reefs are the main habitat of green turtles in the Western South Atlantic and most of the tropical Pacific [23–26], where extended sea grass meadows are scarce [27]. As a result, macroalgae and turf algae, but not seagrasses, represent the bulk of green turtle diet in those regions [24, 28, 29].

On these grounds, Goatley and coworkers [23] hypothesized a relevant role for green turtles in the dynamics of algal communities in the Great Barrier Reef if the populations reached pre-exploitation levels. Conversely, ecosystem modeling suggests that sea urchin grazing is the major determinant of algal cover in Hawaiian reefs, with a relevant role for green turtles only in intertidal rocky habitats [30]. Nevertheless, there is a paucity of data about the abundance of green turtles in tropical reef habitats and little is known about their contribution to the total biomass of herbivores. This paper aims to assess the potential contribution of green turtles to the total herbivore biomass of shallow tropical reefs of oceanic islands in the Western South Atlantic and the Central Pacific Ocean, by means of underwater censuses of green turtles, herbivorous fishes and sea urchins.

## Materials and methods

### Ethics statement

Surveys were conducted according to permits issued by the Instituto Chico Mendes de Conservação da Biodiversidade-ICMBio, Brazil (permit ICMBio/SISBIO 52128–1) and the Department of Land & Aquatic Resources, Hawaii, USA (permit 2019–52). No fishes or sea turtles were captured for the current study. Sea urchin horizontal test diameter was measured on site and specimens were released immediately.

### Study sites

**Fig 1:**
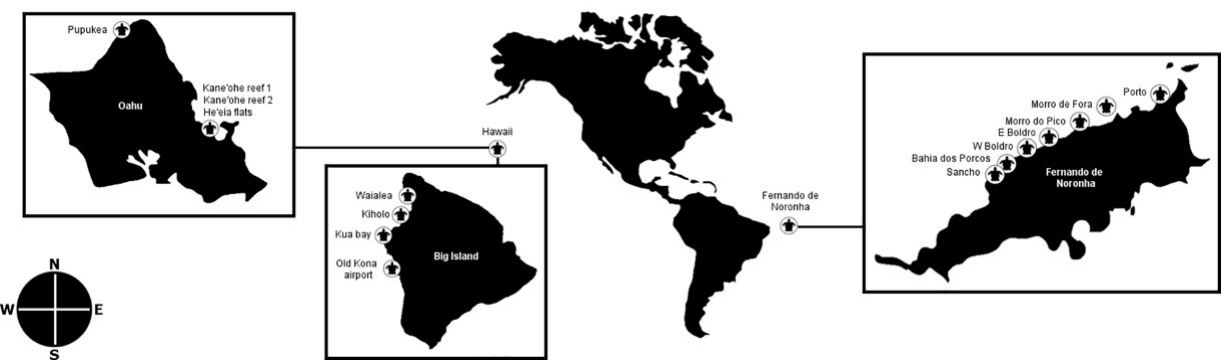
Location of sampling sites in Fernando de Noronha and Hawaii. Map not to scale. Figure is similar but not identical to the original image from Earth Resources Observatory and Science Center and is for illustrative purposes only.

Underwater surveys were conducted by snorkeling in September 2017 at seven sites in Fernando de Noronha (Western South Atlantic Ocean) and September 2018 at eight sites in the islands of Hawaii and Oahu (Hawaiian Archipelago, Central Pacific Ocean) as depicted in Fig 1.

Surveys coincided with the end of the dry season in both areas and were conducted always at high tide. Each site was visited at least twice. The starting point and bearing of each transect (see below) were selected during the first visit to allow evenly spaced transects within the area. The censuses were conducted during the second visit, although sometimes a third visit was required to complete them. The coordinates and characteristics of these sites are detailed in Table 1 and the methods used to asses habitat descriptors are detailed below.

**Table 1:** Major characteristics of sampling sites in the Western South Atlantic Ocean (Fernando de Noronha) and the Central Pacific Ocean (Hawaiian Archipelago) according to the data collected during the present study.

Site	Location coordinates (lat./long.)	Habitat	MPA	Depth (m)	Rugosity index	Live coral (% cover)	Turf (% cover)	Macroalgae (% cover)
**Fernando de Noronha**								
**Porto**	-3.835/32.402	V	Y	1.9 ± 0.5	1.4 ± 0.3	0.0 ± 0.0	19.2 ± 3.1	5.1 ± 2.9
**Morro de Fora**	-3.838/-2.416	RC	Y	1.8 ± 0.7	2.4 ± 0.5	1.0 ± 0.1	19.2 ± 3.5	4.0 ± 2.0
**Morro do Pico**	-3.842/-2.422	V	Y	1.9 ± 0.5	1.3 ± 0.3	0.0 ± 0.0	76.3 ± 1.9	5.9 ± 1.9
**E. Boldro**	-3.844/-2.426	R	Y	1.8 ± 0.7	2.3 ± 0.3	0.0 ± 0.0	44.9 ± 4.4	14.5 ± 5.5
**W. Boldro**	-3.846/-2.429	V	Y	1.8 ± 0.3	1.4 ± 0.3	0.0 ± 0.0	22.3 ± 1.8	2.7 ± 8.9
**Baia dos Porcos**	-3.851/-2.442	RC	Y	1.8 ± 0.6	2.1 ± 0.5	0.2 ± 0.5	24.3 ± 0.7	0.5 ± 0.4
**Sancho**	-3.854/-2.444	RC	Y	2.0 ± 0.6	2.4 ± 0.5	3.2 ± 1.8	18.2 ± 3.6	3.2 ± 1.7
**Hawaiian Archipelago/island of Hawaii-Kona coast**								
**Old Kona Airport**	19.641/-56.008	C	Y	5.0 ± 0.8	3.0 ± 0.2	22.4 ± 16.8	8.0 ± 2.8	0.0 ± 0.0
**Kua bay**	19.810/-56.007	RC	N	3.0 ± 0.4	1.5 ± 0.5	6.0 ± 1.2	13.6 ± 14.4	0.0 ± 0.0
**Kīholo**	19.852/-155.932	RC	N	2.5 ± 0.1	1.0 ± 0.1	31.6 ± 2.8	4.4 ± 5.2	0.0 ± 0.0
**Waialea**	19.981/-155.829	RC	Y	3.0 ± 0.7	2.0 ± 0.5	1.6 ± 1.6	26.8 ± 23.6	0.0 ± 0.0
**Hawaiian Archipelago/island of Oahu**								
**Pūpūkea**	21.656/-158.062	RC	Y	2.0 ± 0.6	2.5± 0.5	13.2 ± 1.2	36.4 ± 30.0	0.0 ± 0.0
**He’eia flats**	21.442/-157.808	CR	N	1.0 ± 0.0	1.0 ± 0.0	0.0 ± 0.0	48.0 ± 10.5	6.2 ± 4.3
**Kāneʻohe reef 1**	21.459/-157.798	C	N	2.5 ± 0.2	3.0 ± 0.2	93.6 ± 3.6	0.0 ± 0.0	6.5 ± 4.5
**Kāneʻohe reef 2**	21.462/-157.798	C	N	2.0 ± 0.3	3.0 ± 0.1	96.4 ± 2.0	0.0 ± 0.0	8.4 ± 4.8

See text for details on methods. Data reported as mean ± standard deviation. Habitat type: vermetid reef (V), rocky reef (R), rocky reef with scattered coral (RC); coral reef (C), coral rubble (CR). MPA (protection from fishing): spear fishing and set nets forbidden (Y), spear fishing and set nets allowed (N).

### Underwater census

Green turtles, herbivorous fishes and sea urchins use habitat at different scales and hence the biomass of each group should be assessed at different scales using different methods [31–34].

Herbivorous fishes were censused visually using four independent and non-overlapping transects of 50 m x 5 m [31–34] parallel to the shore and positioned randomly. Fish were counted on site and only the following truly herbivorous roving fish species were censused: *Kyphosus sectatrix*, *Sparisoma amplum*, *Sparisoma axillare*, *Sparisoma frondosum* and *Sparisoma radians* at Fernando de Noronha [35] and *Acanthurus achilles*, *Acanthurus blochii*, *Acanthurus guttatus*, *Acanthurus leucopareius*, *Acanthurus nigricans*, *Acanthurus nigroris*, *Acanthurus triostegus*, *Calotomus carolinus*, *Calotomus zonarchus*, *Kyphosus spp*., *Naso unicornis*, *Naso lituratus*, *Scarus dubius*, *Scarus perspecillatus*, *Scarus psittacus*, *Scarus rubroviolaceus*, *Scarus sordidus*, *Zebrasoma flavescens* and *Zebrasoma veliferum* at the Hawaiian Archipelago [10, 36–38]. Other species of Acanthuridae rely primarily on detritus or zooplankton. Territorial herbivores (damselfish and blennies) were not considered.

Each fish in the transect was identified to the species level, included in a 5 cm length class, and counted. Fish size was then converted to fish biomass using the equation *weight* = *a* × *length^b^* with a and b values for that species from FishBase [39]. When the total length (TL) was not available, we converted the fork length (FL) to TL. If the weight-length equation was not available for the species, the genus equation was used.

Once the fish were counted, depth was recorded at 0, 10, 20, 30, 40, and 50 m from the starting point of the transect, to calculate the average. Habitat rugosity was assessed using a relative scale ranging from 1 (flat sea bead) to 4 (seabed with large rocks or coral heads). The cover (%) of erect algae, turf-forming alga and live coral and the abundance of sea urchins were measured along the fish transects (roughly at 10, 20, 30, 40, and 50 m from the starting point) using 0.5 x 0.5 m PVC quadrants (25 quadrants per transect). Quadrants were positioned randomly at flat areas contiguous to the 50 m belt delimiting the central part of the transect. All sea urchins found inside each quadrant were measured with plastic calipers (horizontal test diameter without spines) and counted. The horizontal test diameter was converted to biomass following McClanahan [40]. The cover (%) of erect algae, turf-forming alga and live coral within each quadrant was estimated using the internal 25-cell grid of the quadrant [31–34].

Finally, the abundance of green turtles was assessed in four 100 m x 10 m transects parallel to the shore [25, 41–43]. Turtle transects overlapped with those used for fish censuses. Each turtle was counted and was included in a 10 cm length class and its behavior (foraging, resting, swimming) was noted. Carapace length was later converted to biomass using the following equation: *W* = −35.823 + 0.966*CCL*, where *W* is weight in kg and *CCL* is length in cm (R^2^ = 0.887, p<0.001). This unpublished equation has been calculated previously by two of the authors (LC and PC) for juvenile green turtles at the Tamar field station in Ubatuba (Brazil).

### Statistical analysis

The cover of erect algae, turf-forming alga and live coral and the sea urchin biomass at each site is reported as the average of 100 quadrants. Fish biomass and green turtle biomass at each site are reported as the average of four transects. The average biomass of sea urchins, herbivorous fishes and green turtles at each site was expressed as tons per square kilometer (tons/km^2^) to allow comparison [30].

Normality was checked with the Lilliefors test and data were transformed as *log*_10_(*x* + 1) or the *sin*^−1^(*x*) when necessary. MATLAB Simulink Student Suite R2019a was used to analyze the correlations between green turtle, sea urchins and fish biomasses with the environmental descriptors measured at each location (depth, rugosity, coral, algae and turf cover) with the Pearson correlation test. Simple or multiple linear regressions were plotted using a robust bisquare fit to characterize these correlations and find the best model to predict herbivore biomass with those environmental descriptors. Model selection was based on corrected r^2^ and p values (α<0.05). Student’s t-test were used for pairwise comparisons.

## Results

The survey was conducted at sites less than 5 m deep and with a habitat rugosity less than 3, both in the Fernando de Noronha and the Hawaiian Archipelago (Table 1). Depth and rugosity were uncorrelated (p = 0.208, n = 15). Live coral was scarce and covered less than 3.6% of the seafloor in Fernando de Noronha sites. Hawaiian sites were more variable and live coral cover ranged 0–96%. Live coral cover was positively correlated with depth (r = 0.748, p = 0.001, n = 15) and habitat rugosity (r = 593, p = 0.020, n = 15). Turf cover was usually high at Fernando de Noronha (18–76%) and less than 48% at the Hawaiian Archipelago) (Table 1). Macroalgae cover was low in both areas: 0.5–15% at Fernando de Noronha and ≤8% at the Hawaii Archipelago (Table 1).

The most common erect macroalgae species in the quadrants at Fernando de Noronha were *Caulerpa racemosa*, *Dictyopteris plagiogramma* and *Sargassum* spp. *Dictiosphaeria cavernosa* was the only macroalgae observed in the quadrants at the Hawaiian Archipelago, although other species where observed outside the quadrants. Turf and macroalgae cover were pooled for later analysis. Total algae cover was uncorrelated with habitat rugosity (p = 0.181, n = 15) and negatively correlated with depth (r = -0.595, p = 0.019, n = 15) and live coral cover (r = -0.817, p<0.001, n = 15).

**Fig 2:**
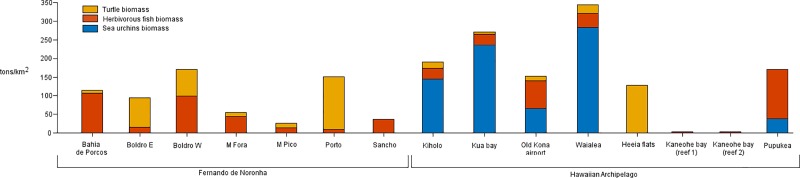
Biomass of herbivores (green turtles, sea urchins and fishes) at reef habitats in Fernando de Noronha and Hawaii.

Sea urchins were virtually absent from Fernando de Noronha, with only two specimens of two different species (*Diadema antillarum* and *Tripnestes ventricosus*) observed, none of them inside any sampling quadrant. Sea urchins, mainly *Echinometra mathaei*, occurred at most sites in the Hawaiian Archipelago, at an average density of 3.7 urchins/m^2^. Nevertheless, sea urchins were absent from the two coral heads and He’eia flats in Kāneʻohe Bay. Sea urchin biomass ranged 0–283.44 tons/km^2^ at the Hawaiian Archipelago (Fig 2) and the best predictor was the model derived from a multiple linear regression including depth and rugosity (R^2^ = 0.608, p = 0.003).

Herbivorous fishes were found everywhere, except at the He’eia flats (Oahu). They were present in very low numbers in the two coral heads in Kāneʻohe. Biomass ranged 13.9–106.6 tons/km^2^ at Fernando de Noronha and 0–132.4 tons/km^2^ at the Hawaiian Archipelago (Fig 2). Fish biomass was uncorrelated with depth (p = 0.204, n = 15), rugosity (p = 0.724, n = 15) or total algal cover (p = 0.550, n = 15). Correlation with live coral cover was marginally significant (r = -0.476, p = 0.073, n = 15).

We observed 103 green turtles in Fernando de Noronha and 47 in the Hawaiian Archipelago. They ranged 40–60 cm and were observed at most sites except Sancho in Fernando de Noronha and Pūpūkea and the two coral heads in Kāneʻohe Bay. One hawksbill turtle (*Eretmochelys imbricata*) was observed at E. Boldro (Fernando de Noronha) but was not considered for later analysis. Green turtles ranging 40–50 cm prevailed in both regions. Green turtles >50 cm were observed only at sites with a rugosity index lower than 1.5. Accordingly, green turtle biomass was negatively correlated with the rugosity index (r = -0.649, p = 0.009, n = 15) and the live coral cover (r = -0.573, p = 0.026). Green turtle biomass was uncorrelated with depth (p = 0.492, n = 15) and total algae cover (p = 0.186, n = 15). Most of the green turtles observed while foraging were grazing intertidal pastures in the Hawaiian Archipelago (intertidal: 24, subtidal: 4), but the opposite was true in Fernando de Noronha (intertidal: 4, subtidal 16).

The biomass of green turtles was uncorrelated with that of herbivorous fishes (p = 0.675, n = 15) and sea urchins (p = 0.653, n = 8 for the Hawaiian Archipelago only). However, the biomass of herbivorous fishes and sea urchins were positively correlated in the Hawaiian Archipelago only (r = 0.839, p<0.001, n = 8). The range of total herbivore biomass was broad in both regions (Fernando de Noronha: 24.2–169.4 tons/km^2^ and the Hawaiian Archipelago: 2.3–342.3 tons/km^2^), but total herbivore biomass was significantly higher in the Hawaiian Archipelago than in Fernando de Noronha (t = -2.71, df = 9, p = 0.024).

In addition, differences existed between the two regions in the contribution of green turtles, fishes and sea urchins to total herbivore biomass (Fig 2). Comparable rocky reef with scattered coral (RC in Table 1) supported a much lower total herbivore biomass (t = 3.4, df = 5, p = 0.017) in Fernando de Noronha (67.7 ± 40.73 tons/km^2^) than in the Hawaiian Archipelago (243.2 ± 78.7 tons/km^2^), likely because of the absence of sea urchins in the former. Indeed, fishes were the major herbivores in most of the rocky reefs with scattered coral off Fernando de Noronha, except for E. Boldro. In contrast, sea urchins were the dominant herbivores in most Hawaiian sites. Green turtles represented less than 8.5% of total herbivore biomass at any Hawaiian site, except in the He’eia flats, where green turtles were the only herbivores present and accounted for a higher biomass (127.7 tons/km^2^). Green turtles were also the dominant herbivores at two sites with a low rugosity index (Porto and W. Boldro) in Fernando de Noronha. Indeed, the contribution of green turtles to the total biomass of herbivores was negatively correlated with the habitat rugosity (r = -0.515, p = 0.05, n = 15) and positively correlated with total algal cover (r = 0.526, p = 0.044, n = 15).

## Discussion

To our knowledge, this is the first study to assess simultaneously the biomass of sea urchins, herbivorous fishes and green turtles in tropical reef habitats. The results reported here suggest that currently the aggregated biomass of fishes or sea urchins make up most of the total herbivore in the reef habitats of tropical oceanic islands. Green turtles make a major contribution to the total herbivore biomass only in flat areas with low live coral cover and high algal cover, a pattern resulting from the contrasting habitat requirements of green turtles and herbivorous fishes and sea urchins. Nevertheless, it should be noted that the biomass of green turtles is much higher than that of any other species of roving herbivore at many sites, although it´s much lower than the aggregated biomass of herbivorous fishes.

The distribution of sea urchins in tropical reefs is strongly determined by water movement, substrate type and sedimentation rate [44–46]. This could explain the virtual absence of sea urchins from the sheltered sites of the Hawaiian Archipelago (the He’eia flats and the two coral heads at Kāneʻohe Bay) and a much higher abundance at deeper, exposed sites (Old Kona Airport, Kua bay, Kīholo, Waialea and Pūpūkea). Sea urchins were virtually absent from all the sampling sites at Fernando de Noronha, as in 1985, when the first comprehensive survey of benthic habitats was conducted [47]. This suggests that the population of *D*. *antillarum* might have collapsed at a similar time to that in the Caribbean [4, 6], although the collapse was unnoticed because of the absence of previous research.

Habitat complexity and wave exposure are the major determinants of fish biomass both in Fernando de Noronha [48] and the Hawaiian Archipelago, although protection from fishing is also relevant in the latter [31, 32, 49, 50]. Most of the sites included in the study were no-take zones, although fishing was allowed at five of the Hawaiian sites (Table 1). Therefore, it is not surprising that the lowest biomass of herbivorous fishes was recorded at those sites open to fishing, thus confounding the role of environmental determinants on the distribution of fish biomass.

In contrast, green turtles are legally protected both in Fernando de Noronha and the Hawaiian Archipelago and hence, patterns of biomass distribution are determined by natural factors such as habitat rugosity and live coral cover. The reasons why green turtles, and particularly specimens >50 cm, concentrate at flat, sheltered areas are unknown, but might be related to food availability and predator avoidance. Morro Pico, in Fernando de Noronha, was the only shallow and flat site where the biomass of green turtles was low, likely because of the strong currents that sweep the area.

Previous studies in oceanic islands across the Central and Western Pacific Ocean reported a patchy distribution of green turtles both at regional and local scales, with sea surface temperature, chlorophyll level and human disturbance as the main drivers [25]. Nutrient availability is one of the major determinants of algal cover and primary productivity in tropical reef systems [51, 52] and hence a higher green turtle biomass was expected at sites with a higher algal productivity. Although green turtle biomass was uncorrelated with total algal cover in the present study, the contribution (%) of green turtles to the total herbivore biomass was positively correlated with total algal cover, which stresses the potential relevance of green turtles as herbivores in areas of enhanced primary productivity.

It should be noted that total algal cover was usually low in subtidal habitats in the Hawaiian Archipelago, likely because of intense sea urchin grazing [30]. The highest algal cover of all the sites surveyed in the Hawaiian Archipelago during this study was recorded at the He’eia flats, an intertidal area covered with coral rubble and devoid of sea urchins and roving herbivorous fishes at high tide. Therefore, it is not surprising that large numbers of green turtles aggregated there at high tide. The existence of a *Halophila* spp. meadow at the nearby Kāneʻohe Sandbar (~1 km away), may facilitate the presence of green turtles there.

Low algal cover in subtidal habitats in the Hawaiian Archipelago may explain why most of the turtles observed while foraging in the Hawaiian sites were scraping turf from intertidal rocks or coral rubble. This behavior has been previously reported at Kaloko-Honokōhau, on the west coast of Hawaii [30], and could be common throughout the Hawaiian Archipelago. In contrast to the situation observed in the Hawaiian Archipelago, green turtles were observed usually foraging in subtidal habitats in Fernando de Noronha. This is likely because of the much higher vegetation cover at subtidal habitats in Fernando de Noronha compared with the Hawaiian Archipelago. Certainly, the distribution of green turtles may vary along the tide cycle and they may forage in subtidal habitats at low tide. But the relevant point here is that green turtles exploited intensely intertidal habitats in the Hawaiian Archipelago when available, but not in Fernando de Noronha. Differences in the algal cover of subtidal habitats in both regions offer the best explanation as to the difference.

Nevertheless, food availability alone cannot explain the preference of green turtles for the flat and sheltered areas of Western Boldro and Porto in Fernando de Noronha, as they have only modest algal cover and high turtle biomass. Sharks (*Carcharhinus perezi* and *Negaprion bervirostris*) were spotted at all the sampling sites in Fernando de Noronha, except Western Boldro, Porto and Morro do Pico. This is evidence that predatory avoidance may explain the preference of green turtles at Western Boldro and Porto. Nevertheless, complex interactions exist between body condition, forage quality and predation risk [22, 53]. It is worth noting that green turtles >60 cm prevail in deeper habitats (~15 m) in the Hawaiian Archipelago [25], which is consistent with reduced predation risk at larger body size. Certainly, further research is required to better understand habitat selection by green turtles in tropical reef habitats in relation to predator avoidance.

In any case, our results show that only sheltered, flat areas with a low coral cover support a high biomass of green turtles in the shallow reefs of tropical oceanic islands. This is an expected result, considering the similarity of those sheltered, flat algal pastures, with the seagrass meadows that represent the favored habitat of green turtles in most of their distribution range [22]. It should be noted that green turtles could be less dependent on these flat algal pastures in the reefs of tropical continental regions, as they usually support a much higher primary algal productivity than the reefs of oceanic islands [51, 52, 54].

Furthermore, coral reefs and seagrass meadows are often intermingled in continental regions, whereas seagrass meadows are poorly developed in most oceanic islands other than atolls [27]. As green turtles can use both seagrasses and algae concurrently [55–57], nearby seagrass meadows may subsidize green turtle population in atolls and continental reef habitats, thus resulting in a much higher green turtle biomass in reef habitats. Further research on habitat use, diet selection, grazing rates and connectivity between seagrass meadows and reefs is required to improve our understanding of green turtle role in the dynamics of underwater vegetation in tropical reef ecosystems.

Finally, the legacy of the past human exploitation of green turtles should be considered, because current population size is probably much lower than in the pre-harvest period, despite population increase in the past decades. The recovery of green turtle populations began worldwide in the 1980´s [16], and has been particularly successful in the Western South Atlantic Ocean and the Hawaiian Archipelago [16–20] Currently, the encounter rate of green turtles in underwater surveys at Fernando de Noronha is twice that reported a decade ago [43], but the pre-harvest population density is unknown. Estimates of past green turtle abundance in the nearby Greater Caribbean suggest that, a decade ago, the population of green turtles was three orders of magnitude lower than their historic numbers [58]. If this was also true for the Western South Atlantic, green turtle density in Fernando de Noronha is still well below carrying capacity and green turtle could have been the dominant herbivores in pre-harvest times, although this is highly speculative.

Green turtles in the Hawaiian Archipelago have one the lowest population densities reported from oceanic islands in the Central and Western Pacific Ocean, due to a combination of low sea surface temperature, low primary productivity and high human impact [25]. Nevertheless, the green turtle population in the Hawaiian Archipelago exhibits a high growth rate thanks to legal protection [16, 17, 25] and green turtles have been close to carrying capacity on the west coast of the island of Hawaii for more than two decades [59]. Ecosystem modeling indicates that the dynamics of subtidal vegetation at Kaloko-Honoköhau, on the west coast of the island of Hawaii, is ruled by sea urchin grazing, with green turtles playing a relevant role only at intertidal rocky habitats [30]. Considering the similarity in the biomass and make-up of the herbivore community at Kaloko-Honoköhau and the other sites analyzed in this study, green turtles are likely to play a minor role in the dynamics of subtidal vegetation in these places. Further increase in green turtle biomass would be unlikely in that part of the Hawaiian Archipelago if the green turtle population there is approaching carrying capacity [59], and hence, sea urchins and roving herbivorous fishes will likely continue to dominate the herbivore biomass on the west coast of the island of Hawaii in the future.

The situation could be different at more productive sites, such as Kāneʻohe Bay, in Oahu, one of the most productive areas in the Hawaiian Archipelago [60]. Green turtles exhibit a much higher rate of somatic growth there than on the west coast of the island of Hawaii [59] and the algal pastures at the He’eia flats support the highest green turtle biomass reported in this study for the Hawaiian Archipelago. There is no evidence that green turtles have reached carrying capacity at Kāneʻohe Bay [59], and hence, biomass could increase in the future in areas with a low cover of live coral. Nevertheless, nothing can be said about the original situation in pre-harvest times, due to the dramatic modification of the bay during the 20th century.

Finally, a better understanding of trophic redundancy within the community of herbivores is required to assess the role of green turtles in tropical reef habitats. This study shows that green turtle biomass is often much smaller than the aggregated biomass of herbivorous fishes or sea urchins. Furthermore, the latter two groups have much higher daily feeding rates than green turtles: 29% of body weight for parrotfish and surgeonfish [61] and 13% of body weight for the sea urchin *E*. *mathaei* [62], compared to <1% of body weight for green turtles [21]. Nevertheless, green turtles may still play a relevant role in the dynamics of the underwater vegetation of tropical reefs if they used resources differently from fishes and sea urchins. This is because different species may target different algal resources, thus creating the potential for strong complementarity in reef habitats, where more species might be needed to maintain ecosystem functioning across large, naturally varied reefscapes than suggested by small-scale studies [7, 33, 50]. However, this kind of data is not yet available, so in in order to gain further insight into these specific questions, further research is needed.
